# Influence of Gut Microbiota on Immune Responses and Protection in Volunteers Receiving the Live Attenuated Oral ETEC Vaccine ACE257 followed by Virulent ETEC H10407 Challenge

**DOI:** 10.21203/rs.3.rs-9284363/v1

**Published:** 2026-05-04

**Authors:** Ethan Gough, Soumya Basu, Jessica Brubaker, Barbara DeNeraing, David Sack, A Louis Bourgeois, Richard Walker, Clayton D. Harro, Subhra Chakraborty

**Affiliations:** Bloomberg School of Public Health, Johns Hopkins University; Bloomberg School of Public Health, Johns Hopkins University; Bloomberg School of Public Health, Johns Hopkins University; Bloomberg School of Public Health, Johns Hopkins University; Bloomberg School of Public Health, Johns Hopkins University; PATH; PATH; Bloomberg School of Public Health, Johns Hopkins University; Bloomberg School of Public Health, Johns Hopkins University

**Keywords:** ETEC, Diarrhea, vaccine, microbiome, immune response, sequencing

## Abstract

Enterotoxigenic Escherichia coli (ETEC) remains a major cause of diarrheal morbidity with no licensed vaccines. Role of gut microbiota in vaccine immunogenicity and protection was investigated using 16S rRNA sequencing from the stool samples of 27 volunteers receiving two doses of the live attenuated oral ETEC vaccine ACE527 followed by virulent ETEC H10407 challenge. Systemic and mucosal IgG and IgA responses to heat-labile toxin-B (LTB) and colonization-factor-antigen-I (CFA/I) were quantified by ELISA in serum and antibody-in-lymphocyte-supernatant (ALS). Microbiome α-diversity, β-diversity, and taxa–immune associations were evaluated using regression models, MiRKAT, and relaxed LASSO. Vaccination increased (~ 25–30%) Eubacterium_brachy_group, Family_XIII_AD3011 and Actinomyces. Higher α-diversity (inverse-Simpson) was associated with reduced ALS anti-LTB and CFA/I IgA responses, whereas β-diversity correlated with increased serum anti-CFA/I IgA. Members of Anaerovoraceae, Peptostreptococcaceae, Oscillospiraceae, and Veillonellaceae enhanced immune responses and protection against severe diarrhea and ETEC colonization, while Ruminococcaceae, Sutterellaceae, Coriobacteria, Clostridia, and Actinobacteria showed antagonistic associations.

## Introduction

Enterotoxigenic *Escherichia coli* (ETEC) diarrhea and associated morbidity and mortality remain a significant global health concern, especially among children and travelers, particularly in low- and middle-income countries^1,2^. ETEC causes an estimated 220 million diarrhea episodes globally, with approximately 75 million episodes occurring in children under 5 years of age. The mortality estimates for children under 5 years range from 18,700 to 42,000 deaths annually^3^. The development of effective vaccines against ETEC has been a long-standing priority for the WHO, with the potential to significantly reduce the burden of diarrheal disease^4,5^.

The human gut microbiota, a complex ecosystem of microorganisms residing in the gastrointestinal tract, plays a crucial role in shaping the host immune response. Recent advances in microbiome research have revealed the intricate relationships between gut bacteria and various aspects of human health, including immune function and vaccine efficacy^6–8^. The gut microbiota has been shown to modulate both innate and adaptive immune responses, influencing the development and function of immune cells, cytokines and antibody production, and the host’s overall immune landscape^8^.

Vaccines, specifically administered orally, must navigate the complex microbial environment of the gut to elicit an effective immune response. The interplay between the vaccine strains, the resident microbiota, and the host immune system is multifaceted and can impact vaccine efficacy. Recent studies have highlighted the importance of considering gut microbiota in vaccine development and evaluation^9^. For instance, polio and rotavirus vaccines have shown that differences in gut microbiota composition can influence vaccine efficacy^10^. We previously showed in a human ETEC experimental challenge model that specific gut flora is predictive of post-challenge diarrhea outcomes^11^. Understanding these relationships is crucial for optimizing vaccine design and predicting vaccine outcomes in diverse populations. However, evidence for associations among the gut microbiome, immune responses, and the efficacy of enteric vaccines such as ETEC remains limited.

**Figure 1:**
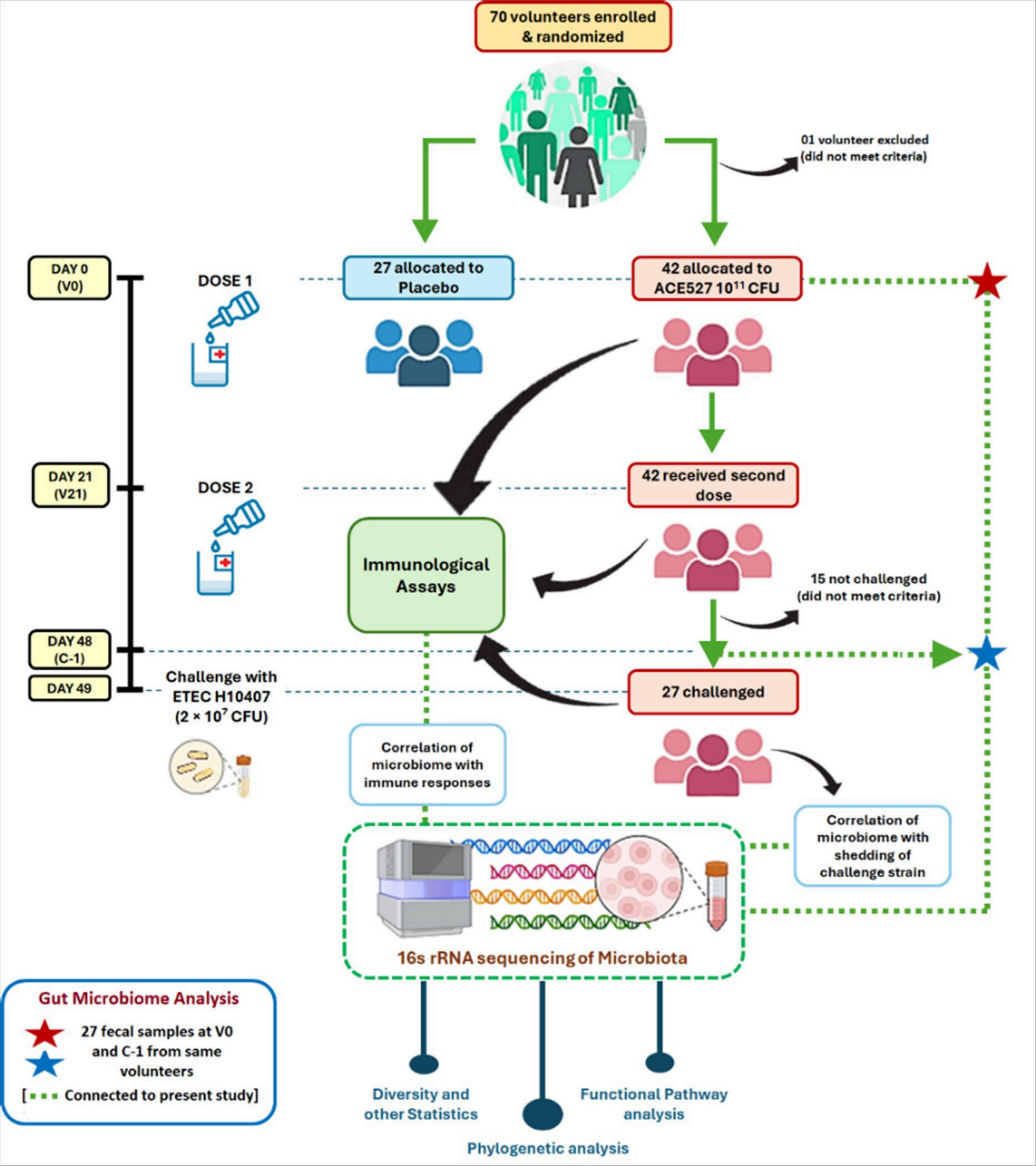
Overall scheme of the vaccine trial with focus on the microbiome study

In this study (Fig. 1), we aimed to determine the changes in gut microbiome following immunization and the potential associations between gut microbiome, vaccine-induced immunity, and vaccine efficacy using a randomized clinical trial where volunteers were immunized with a candidate oral ETEC vaccine ACE527 and subsequently challenged with the virulent ETEC strain H10407.

## Results

### Does vaccination change the gut microbiome?

#### Specific microbiome changes following vaccination (between V0 and C-1)

McNemar’s test for paired data indicated three taxa differed in presence or absence between V0 and C-1. Eubacterium_brachy_group increased from among 3.7% of participants at V0 to 33.3% of participants at C-1 with a p-value of 0.027, alongside two other groups, Family_XIII_AD3011 and Actinomyces, both detected in 3.7% of participants at V0 and 29.6% at C-1 with p-values of 0.046.

#### Changes in gut microbiome α-diversity between V0 and C-1

The analysis of α-diversity reveals a relatively stable gut microbiome structure between V0 and C-1. The Shannon index, inverse Simpson index, and Pielou’s evenness all showed slight increases (< 10%) in diversity from V0 to C-1, but none of these changes were statistically significant (p-values ranging from 0.335 to 0.757). (Table 1).

#### Changes in gut microbiome β-diversity between V0 and C-1

Within-subject β-diversity between V0 and C-1 was significantly greater than zero. The median Bray-Curtis dissimilarity of 0.28 indicated a moderate level of similarity in genus composition and abundance between V0 to C-1 microbiomes (p < 0.001) with a narrow interquartile range (0.21 to 0.32) (Table 2). On a similar note, the median Jaccard similarity of 0.44 indicated moderate overlap in genus detection between microbiomes, with an average 44% dissimilarity (p < 0.001).

#### How does the microbiome correlate with the vaccine-induced immune responses?

The vaccine immune responses associations with the microbiome were depicted by estimating mean of antibody fold changes (normalized to a Gaussian distribution) per log-scale shifts in microbiome diversity indices using linear regression. We analyzed the primary vaccine antigens CFA/I and LTB, which are also present in the challenge strain and have the potential to be protective.

#### LTB antibody fold changes per log_2_ increase in microbiome α-diversity

**Figure 2:**
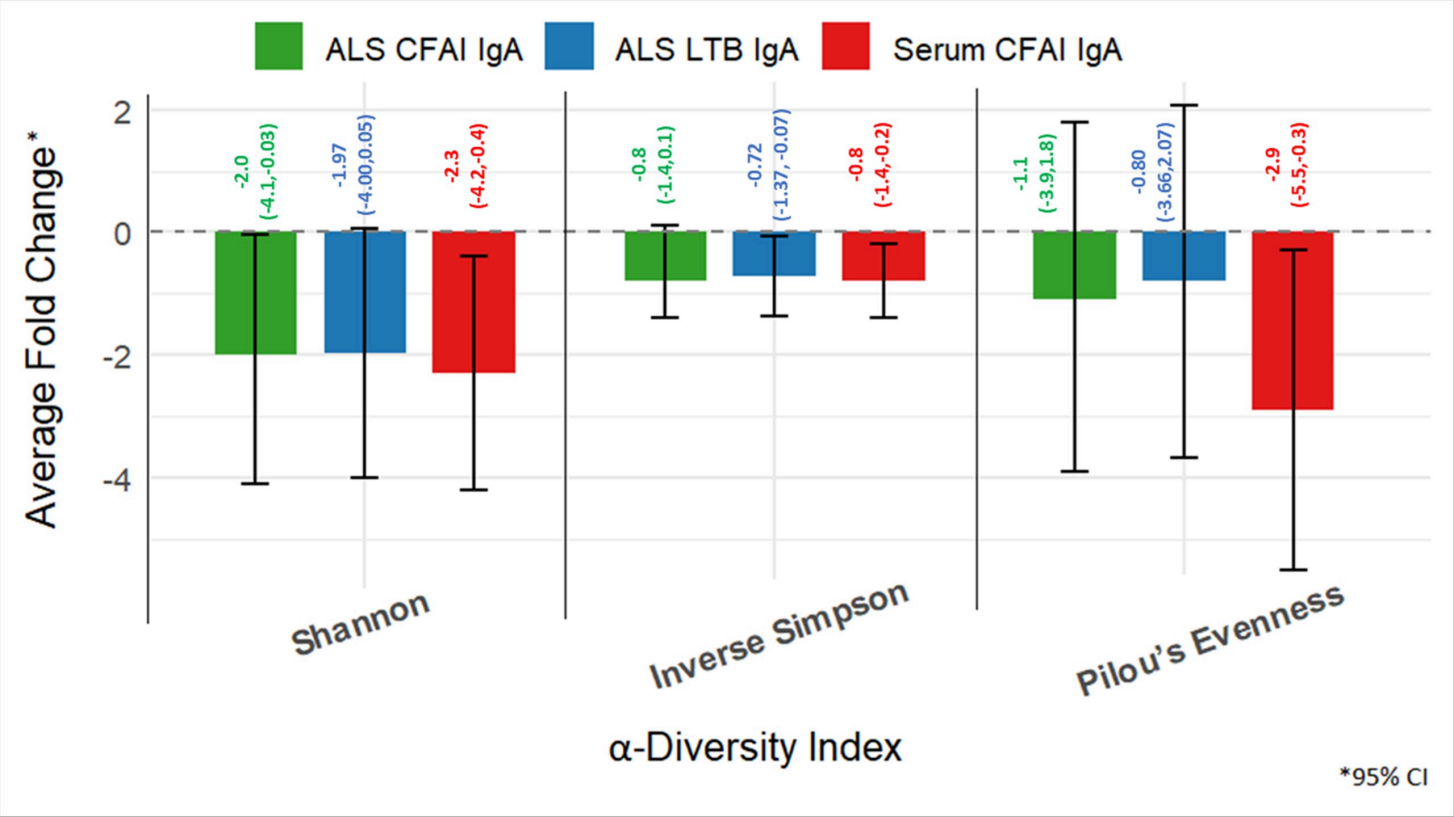
ALS LTB, ALS CFA/I and Serum CFA/I IgA Fold Changes per log_2_ increase in α-diversity (V0 to V21) [antibody titers between two timepoints were transformed into fold changes and normalized to a Gaussian distribution].

The analysis of average fold change in LTB IgA levels in ALS between V0 and V21 per log_2_ increase in α-diversity revealed a statistically significant negative association with inverse Simpson diversity, where each log_2_ increase in inverse Simpson diversity was associated with a 0.72 (95%CI: −1.37, −0.07, p = 0.037) decrease in ALS LTB IgA V0 to V21 fold change. The Shannon index showed a near statistically significant inverse relationship (−1.97; 95% CI: −4.00, 0.05, p = 0.064) (Fig. 2). A similar trend of negative association with α-diversity indices was observed for fold change of LTB IgA (V0 to V28) in ALS, and LTB IgA or IgG fold change in serum (V0 to V21 and V0 to V31) however, they were not statistically significant.

#### CFA/I Antibody fold Changes per log increase in microbiome α-diversity

Average fold change in CFA/I IgA from V0 to V21 in ALS per log_2_ increase in α-diversity was also negatively associated with the inverse Simpson index (−0.8, 95% CI: −1.4,0.1, p = 0.023) and showed a near statistically significant inverse association with the Shannon index (−2.0, 95%CI: −4.1, −0.03, p = 0.055). Similarly, CFA/I IgA responses from V0 toV21 in serum suggest that a per log_2_ increase in α-diversity was associated with reduced IgA fold change (p < 0.05) (Fig. 2). Similar trends were observed between V0 and V31 CFA/I antibody fold increase, but the association was not statistically significant. (**Supplementary File 1**). Fold Change of CFA/I IgG in serum from V0 to V21 and V0 to V31 per log_2_ increase in α-diversity was not statistically significant and did not show any distinct trend.

#### LTB antibody fold changes with an increase in β-diversity

Fold change from V0 to V21 and V0 to V31 of LTB IgA titers in serum did not show a significant association with β-diversity. The Bray-Curtis and Jaccard indices showed considerable compositional scatter, with no evidence of association with LTB IgA fold changes (p > 0.05) in serum. Similarly, β-diversity analyses of ALS LTB IgA fold change V0 to V21 and V0 to V28 indicated no associations.

#### CFA/I antibody fold changes associated with increase in β-diversity

**Figure 3:**
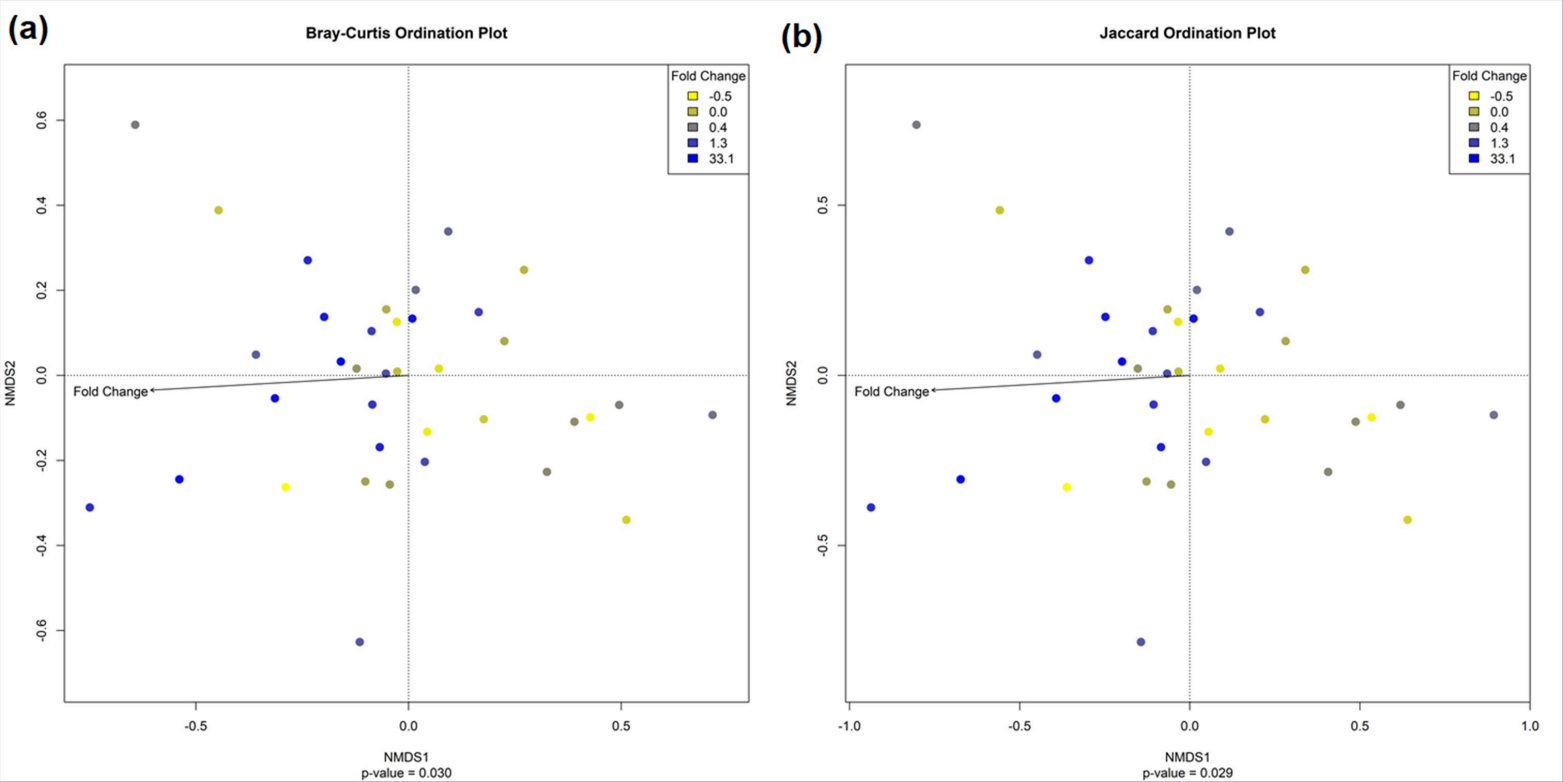
**(a)** Bray-Curtis β-diversity analyses by fold increase of CFA/I IgA antibody titers from V0 to V21 in serum, **(b)** Jaccard β-diversity analyses by fold increase of CFA/I IgA from V0 to V21 in serum. Arrows parallel to the non-metric multidimensional scaling (NMDS) axis are perfectly correlated with that axis scores, indicating that variability in microbiome composition represented by that axis is associated with antibody fold change [antibody titers between two timepoints were transformed into fold changes and normalized to a Gaussian distribution]. Colors, yellow to dark blue, indicate an increasing antibody fold change. p-values were produced using MiRKAT.

A greater variability in microbiota composition (β-diversity) was associated with larger fold change in CFA/I IgA from V0 to V21 in serum [MiRKAT p = 0.030 with Bray-Curtis and MiRKAT p = 0.029 with Jaccard] (Fig. 3).

There was no uniformly observable trend or statistically significant association (p > 0.05) between CFA/I IgG fold change from V0 to V21 or V0 to V31 and β-diversity using either Bray Curtis or Jaccard dissimilarity indices. ALS CFA/I IgA fold change also showed no significant associations with β-diversity for either the V0 to V21 or V0 to V28 periods (MiRKAT p > 0.05).

#### Predictors of fold changes in LTB antibody titers following vaccination

**Figure 4:**
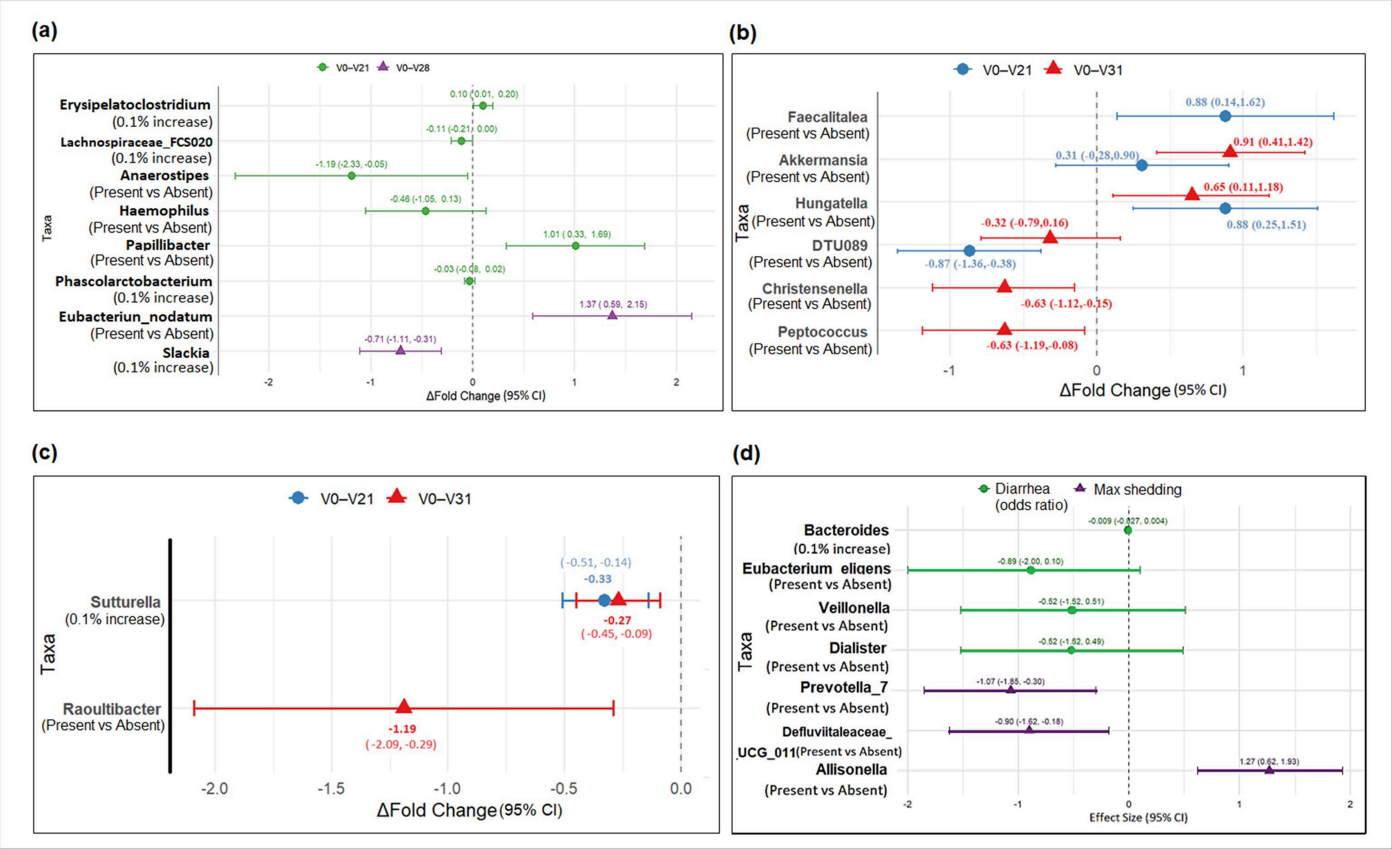
Associations between antibody fold change and selected taxa estimated by linear regression. Selected taxa were chosen using the relaxed LASSO to identify key predictors. The normalized average antibody fold change associated with the ‘presence’ and/or ‘per 0.1% increase’ in relative abundance of the genus are indicated in parentheses below taxon. **(a)** Key predictors of ALS LTB IgA fold change (V0 to V21 & V0 to V28) **(b)** Key predictors of serum CFA/I IgA fold change (V0 to V21 & V0 to V31) **(c)** Key predictors of serum CFA/I IgG fold change (V0 to V21 & V0 to V31) **(d)** Key predictors for moderate-to-severe-diarrhea (MSD), and max shedding

Between V0 to V21, a group of six bacterial taxa were identified by relaxed LASSO regression that predicted fold increase in LTB IgA in ALS titers, including Erysipelatoclostridium, Lachnospiraceae_FCS020_group, Anaerostipes, Haemophilus, Papillibacter, and Phascolarctobacterium. Presence of the bacterial genus Papillibacter was associated with an increased fold change of 1.01 (95%CI: 0.33,1.69; adjusted p = 0.028) (Fig. 4a). In contrast, a 0.1% increase in relative abundance of Erysipelatoclostridium was associated with a 0.10 (95% CI:0.01,0.20, p = 0.032) increase of LTB IgA antibody titers in ALS, but it was not statistically significant after correction for multiple testing (**Supplementary File 1**). Similarly, two bacterial taxa, Slackia and Eubacterium nodatum group, were identified as predictors of LTB IgA fold increase from V0 to V28 in ALS (Fig. 4a). A 0.1% increase in the relative abundance of Slackia was associated with − 0.71 (95%CI: −1.11, −0.31; adjusted p = 0.002) decrease in fold change, while the presence of Eubacterium_nodatum_group was associated with a 1.37 (95%CI:0.59,2.15; adjusted p = 0.002) increase in fold change (**Supplementary File 1**). No significant specific taxon associations were found for LTB IgA or IgG fold changes in serum.

#### Predictors of fold change in CFA/I antibody titers following vaccination

Twenty-five taxa were selected as predictive of CFA/I IgA V0 to V28 fold change in ALS although Erysipelotrichaceae_UCG_003, Eubacterium_nodatum_group, Merdibacter, Terrisporobacter, Actinomyces, Ruminococcus_gauvreauii_group, Lachnospiraceae_UCG_002 and Sutturella were statistically significant. Each 0.1% increase in Erysipelotrichaceae_UCG_003 and the presence of Merdibacter, Ruminococcus_gauvreauii_group, and Lachnospiraceae_UCG_002 were associated with an increased CFA/I IgA fold change of 0.07 (95%CI: 0.05,0.08; p < 0.001), 0.47 (95%CI: 0.04,0.90;p = 0.033), 0.46 (95%CI: 0.05,0.88; p = 0.031) and 0.43 (95%CI: 0.02,0.85, p = 0.041), respectively, but only Erysipelotrichaceae_UCG_003 remained statistically significant after correction for multiple testing. Conversely, each 0.1% increase in Sutturella, and the presence of Eubacterium_nodatum_group and Actinomyces were associated with a decreased fold change of CFA/I IgA, − 0.19 (95%CI: −0.32,−0.06;p = 0.008), −0.48 (95CI: −0.96,−0.01; p = 0.045) and − 0.47 (95%CI: −0.92,−0.02; p = 0.043), respectively, but none were statistically significant after adjustment of multiple testing (**Supplementary File 1**). No significant specific taxon associations could be found for CFA/I IgA fold changes in ALS.

Eight and fifteen bacterial taxa were selected as predictors of CFA/I IgA fold changes in serum between V0 to V21 and V0 to V31, respectively (**Supplementary File 1**). Between V0 to V21, presence of DTU089 was associated with a −0.87 (95%CI: −1.36,−0.38; adjusted p = 0.009) decreased fold change, while presence of Faecalitalea and Hungatella were associated with a 0.88 (95%CI: 0.14,1.62, p = 0.021) and 0.88 (95%CI: 0.25,1.51, p = 0.008) fold increase that were not statistically significant after adjustment of multiple testing (adjusted p ≥ 0.05). From V0 to V31, presence of Hungatella was also associated with a 0.65 (95%CI: 0.11.1.18; p = 0.020) increased fold change, but it was not statistically significant after adjustment for multiple testing (adjusted p = 0.264). Other taxa identified as predictors of serum IgA V0 to V31 increased fold change were Akkermansia (0.91, 95%CI:0.41,1.42; p = 0.001), and taxa associated with decreased fold change were Christensenella (−0.63,95%CI: −1.12, −0.15; p = 0.013) and Peptococcus (−0.63, 95% CI:−1.19,−0.08,p = 0.027) (Fig. 4b). However, only Akkermansia was statistically significant after adjustment for multiple testing (adjusted p = 0.016) (**Supplementary File 1**). However, Akkermansia, Libanicoccus, and DTU089 were also identified as important predictors of CFA/I IgA fold change in serum in both periods V0 to V21 and V0 to V31 but were not statistically significant as independent predictors.

The CFA/I IgG titer fold change in serum between V0 and V21 was inversely associated with Sutturella. A 0.1% increase in relative abundance of Sutterella was associated with a −0.33 (95%CI: −0.51, −0.14, adjusted p = 0.001) decreased fold change. Furthermore, between V0 and V31, Raoultibacter and Sutterella were associated with a lower fold change in CFA/I IgG antibody titers. Presence of Raoultibacter and 0.1% increase in relative abundance of Sutturella in serum were associated with CFA/I IgG fold change of −0.27 (95%CI: −0.45, −0.09, adjusted p = 0.010) and − 1.19 (95%CI: −2.09, −0.29, adjusted p = 0.011) respectively (Fig. 4c).

### How does the day before challenge microbiome correlate with protection from diarrhea following challenge with virulent ETEC H10407 strain?

#### Predictors of MSD

At C-1, Bacteroides, Eubacterium_eligens_group, Veillonella and Dialister were selected by relaxed LASSO as predictors of MSD (not protective) after challenge with ETEC H10407. However, no individual genus was shown to be significantly associated with MSD, which might be due to the small sample size.

#### Predictors of shedding of the challenge strain following ETEC challenge

Prevotella_7, Defluviitaleaceae_UCG_011 and Allisonella were selected as important predictors of shedding of the challenge strain in stool following challenge with ETEC H10407. The presence of Allisonella was associated with a 1.27 log_10_ increase in maximum shedding (95%CI: 0.62,1.93, adjusted p = 0.006) compared to the absence of Allisonella, while the presence of Prevotella_7 and Defluviitaleaceae_UCG_011 were associated with − 1.07 (95%CI: −1.85,−0.30, adjusted p = 0.026) and − 0.90 (95%CI:−1.62,−0.18, adjusted p = 0.026) log10 decrease in max shedding. (Fig. 4d).

There were no significant associations between α-diversity or β-diversity measures at C-1 and MSD, maximum shedding, or length of shedding following challenge. No taxa were selected as strong predictors of the length of shedding by relaxed LASSO regression.

#### Phylogenetic analysis reveals beneficial and harmful taxa

**Figure 5:**
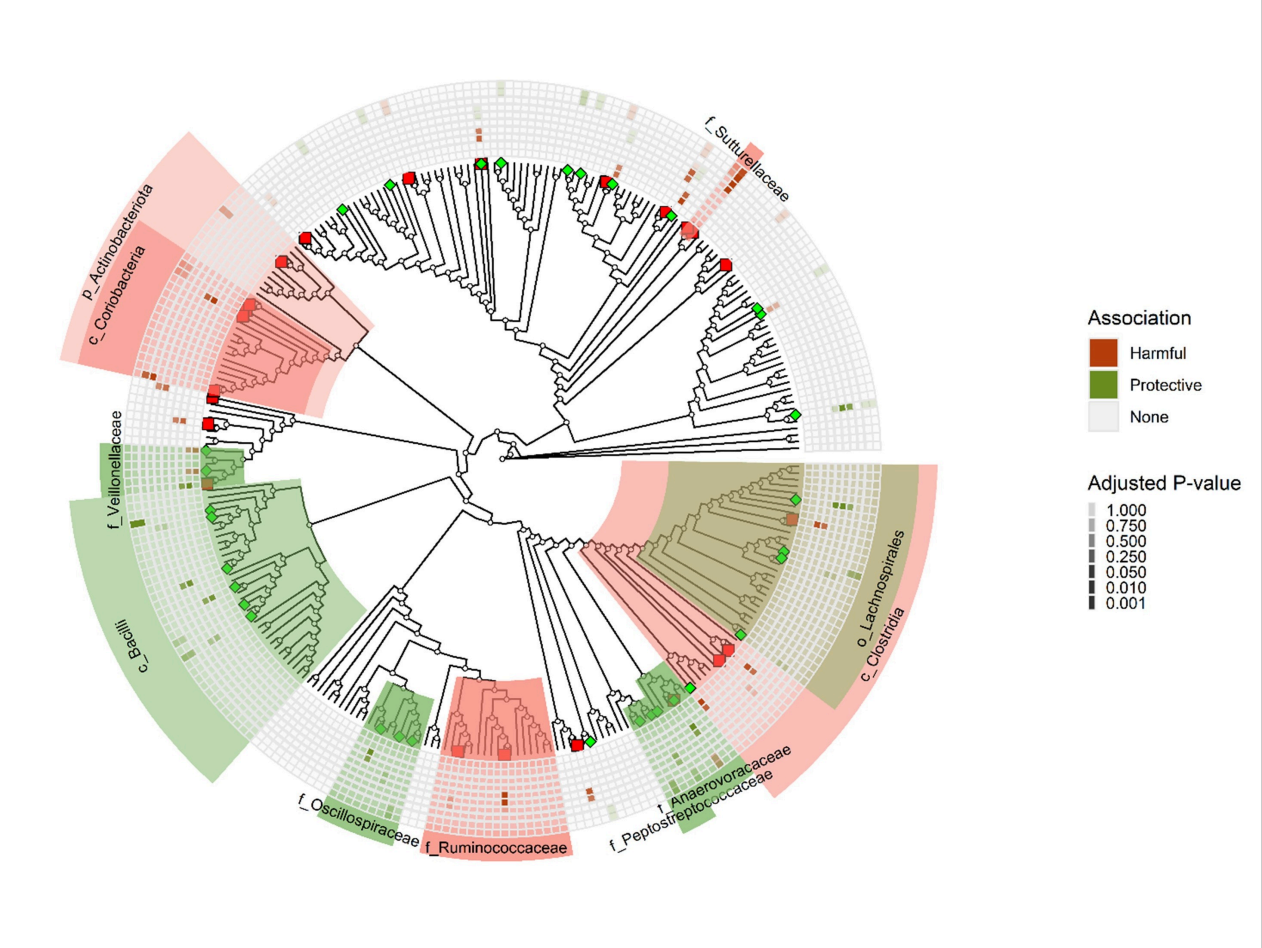
Phylogenetic tree summarizing the specific taxa selected by the relaxed LASSO as predictors of diarrhea, max shedding, or antibody fold change. Tree tips are genus-level taxa. Shaded areas are phylogenic clades. Rings around the tree, from innermost to outermost ring, indicate whether a genus was associated with diarrhea (inner most), max shedding, ALS LTB IgA V0 to V21 and V0 to V28, serum CFA/I IgA V0 to V21 and V0 to V31, serum CFA/I IgG V0 to V21, and V0 to V31 or ALS CFA/I IgA V0 to V28 (outermost circle). Colored tiles in each ring range from light (adjusted p=1.000) to dark (adjusted p≤0.001). Colors of tree tips and shaded areas are green if taxa predicted decreased MSD, reduced max shedding or increased antibody fold change (protective associations) and are red if taxa predicted increased MSD, increased max shedding or length of shedding or decreased antibody fold change (harmful associations).

Visualization of the phylogenetic relationships between taxa associated with various outcomes (diarrhea and shedding) or vaccine antibody responses (Fig. 5) shows that despite the diversity of these genera, they belonged to a handful of higher taxonomic groups (family, class, or order). Genera within the families Anaerovoraceae, Peptostreptococcaceae, Oscillospiraceae, Veillonellaceae, and the class Bacilli were associated with protective effects of the ACE527 ETEC vaccine, as evidenced by their correlations with reduced odds of MSD, decreased challenge ETEC strain colonization (shedding), and enhanced antibody responses following vaccination. Conversely, taxa from the families Ruminococcaceae and Sutterellaceae, the classes Coriobacteria and Clostridia, and the phylum Actinobacteria were linked to failure of the ACE527 ETEC vaccine to protect the host from ETEC colonization and diarrhea, including association with higher odds of MSD, increased ETEC shedding, and diminished vaccine-induced antibody titers. Notably, within the class Clostridia, certain members of the family Lachnospiraceae demonstrated protective associations, underscoring the functional diversity that exists even within taxonomic groups that had detrimental effects.

#### Prediction of potentially significant microbial pathways connected to antibody responses

To gain insight into biological mechanisms that may contribute to the ability of taxa to predict outcomes or antibody responses associated with ACE527 vaccine, we identified PICRUSt2-predicted MetaCyc pathways that differentiate harmful (not protective) and protective taxa. We then performed overrepresentation analyses to determine whether pathways that differentiated protective or harmful taxa were more likely to be involved in specific MetaCyc pathway types. Thirty-six pathways were determined to be more or less frequent among protective or harmful taxa. Among these pathways, taxa that predicted protection from MSD were more likely to carry pathways involved in polymeric compound degradation or glycan pathways, and those that predicted increased serum CFA/I IgA antibody titers from V0 to V31 were more likely to carry pathways involved in amino acid biosynthesis. In contrast, taxa that predicted decreased serum CFA/I IgA antibody titers from V0 to V21 or V31 were less likely to be involved in C1 compound utilization and assimilation and were less likely to be involved in carbohydrate biosynthesis. However, only pathways involved in amino acid metabolism among taxa that predicted increased serum CFA/I IgA antibody titers V0 to V31 showed significant overrepresentation after FDR-adjustment [including L-methionine biosynthesis III, superpathway of L-methionine biosynthesis (by sulfhydrylation), superpathway of L-methionine biosynthesis (transsulfuration)].

Conversely harmful taxa that predicted decreased LTB IgA antibody titers in ALS from V0 to V21 or decreased serum CFA/I IgG antibody titers from V0 to V31 were also more likely to carry pathways involved in amino acid biosynthesis (these pathways were different from L-methionine biosynthesis); while those that predicted reduced ALS CFA/I IgA antibody titers from V0 to V28 were more likely to carry pathways involved in cell structure biosynthesis and antibiotic resistance or nucleoside and nucleotide biosynthesis. While harmful taxa, which predicted decreased serum CFA/I IgG V0 to V21 or reduced serum CFA/I IgG antibody titers V0 to V31 were less likely to carry pathways involved in fatty acid and lipid biosynthesis and those that predicted reduced ALS CFA/I IgA V0 to V28 were less likely to carry pathways involved in amino acid metabolism. However, none of these pathways showed statistically significant overrepresentation after FDR-adjustment. For specific pathways that were more or less likely to be carried by protective or harmful taxa, and which genera carried these pathways, see **Supplementary File 2**.

## Discussion

This study, using an oral live ETEC vaccine-challenge clinical trial, showed that the microbiota can influence vaccine-induced immunity and vaccine efficacy. Among ACE527 vaccine recipients, although the overall microbiome remained relatively stable, measurable changes were observed that were associated with mucosal and systemic vaccine-induced immunity and protection against MSD. This study also identified taxa that are predictive of vaccine efficacy.

In this study, gut microbiome α-diversity remained largely stable between pre-vaccination (V0) and pre-challenge (C-1), with only minor, non-significant changes (median Shannon index: 2.99 to 3.03). This structural stability is notable, as major microbiome disruptions have been linked to altered vaccine responses in prior studies^12^. Despite this overall stability, Eubacterium_brachy_group, Family_XIII_AD3011, and Actinomyces showed marked increases in detection between V0 and C-1, suggesting targeted microbial shifts that are associated with vaccine efficacy or immune modulation ^9,13,14^. α-diversity indices were inversely associated with mucosal antibody responses. Specifically, higher inverse Simpson diversity was significantly associated with lower fold increases in ALS LTB- and CFA/I-specific IgA titers, with a similar trend observed for the Shannon index. These findings suggest that greater microbial diversity may dampen vaccine-induced mucosal antibody responses, consistent with prior reports implicating competitive exclusion of immunostimulatory taxa^15^. Within-subject β-diversity changes between V0 and C-1 was moderate but significant, with a median Bray–Curtis dissimilarity of 0.28 and a median Jaccard similarity of 0.44 (p < 0.001), indicating measurable compositional shifts despite overall stability. Associations between β-diversity and antibody responses were antigen-specific. No significant relationships were observed for LTB-specific IgA responses; however, CFA/I-specific IgA fold changes in serum from V0 to V21 were positively associated with β-diversity, as demonstrated by MiRKAT analyses (Bray–Curtis p = 0.030; Jaccard p = 0.029). This suggests that microbiome community configuration, rather than overall diversity, influenced CFA/I-specific immune responses. Overall, these findings align with broader literature indicating that both α- and β-diversity shape vaccine outcomes, where lower α-diversity may favour stronger responses, while greater β-diversity reflects a dynamic microbiome capable of modulating immune priming and vaccine-induced protection^16^. In our previous study with ETEC H10407 experimental human challenge model, we identified 32 operational taxonomic units (OTUs) that could predict ETEC challenge outcomes, with 12 OTUs associated with resistance to diarrhea, despite individual variations^17^. The findings suggested that certain bacterial taxa, such as *Prevotella copri* and *Bacteroides* species, may play a protective role against ETEC colonization, offering potential targets for probiotic development^17^. We found consistent data in the current study that Bacteroides and Prevotella_7 predicted reduced odds of MSD and reduced shedding, respectively, in the vaccinated group, but only the Prevotella_7 results were statistically significant.

This study highlights a strong link between gut microbiome composition, its functional capacity, and ACE527 vaccine–induced immunity, underscoring the microbiome’s dual role in promoting or impairing protection against MSD. Taxa associated with protection including Anaerovoraceae, Peptostreptococcaceae, Oscillospiraceae, Lachnospiraceae, Veillonellaceae, and the class Bacilli are largely obligate anaerobes that produce short-chain fatty acids (SCFAs) such as acetate, propionate, and butyrate. SCFAs support gut barrier integrity, suppress inflammation, and modulate immune responses, providing a mechanistic explanation for their association with reduced diarrhea severity and enhanced vaccine-induced antibody responses^14,18–24^. Consistent with prior studies, Lachnospiraceae abundance correlated with improved responses to oral vaccines^25^. Veillonella species further enhance mucosal immunity by converting lactate into weaker acids that limit pathogen colonization, while Bacillus species exert immunomodulatory effects and promote beneficial microbial interactions^26,27^. Together, these taxa create a gut environment favourable for effective immune priming. In contrast, Ruminococcaceae, Sutterellaceae, Coriobacteriia, Clostridia, and Actinobacteria were associated with adverse outcomes, including increased risk of diarrhea or reduced vaccine efficacy^28^. Taxa such as Intestinibacter, Faecalitalea, Erysipelotrichaceae_UCG_003, and Sutterella predicted lower CFA/I-specific IgA and IgG responses. Sutterella, in particular, has been linked to increased pathogen shedding and degradation of secretory IgA, weakening mucosal defences and facilitating pathobiont persistence^29,30^.

Additional predictors of diarrhea included Bacteroides, Eubacterium_eligens_group, Veillonella, and Dialister. While often commensal, Bacteroides can become pathogenic under dysbiosis, and Veillonella and Dialister are associated with intestinal inflammation^31–34^. Notably, Allisonella correlated with increased pathogen shedding, suggesting impaired clearance or enhanced replication. Collectively, these findings emphasize the context-dependent influence of microbiome balance on vaccine responsiveness and disease susceptibility.

Functional pathway analyses provided mechanistic insight into these associations. Taxa linked to reduced MSD or enhanced antibody responses exhibited depletion of MetaCyc pathways involved in carbohydrate and cell-surface biosynthesis, including reduced UDP-N-acetyl-D-glucosamine and *E. coli* O-antigen building block pathways^35–37^. This suggests diminished peptidoglycan and surface polysaccharide synthesis, potentially limiting immune evasion and enhancing exposure of innate immunostimulatory ligands such as lipid A^38^. Beneficial taxa were also enriched for amino acid biosynthesis pathways, particularly L-methionine biosynthesis, which correlated with higher CFA/I IgA responses and may support T helper cell activation under conditions of limited dietary methionine^39,40^. In contrast, taxa associated with increased MSD or reduced antibody responses showed enrichment of peptidoglycan, amino acid, and lipid biosynthesis pathways, potentially skewing immunity toward NOD1/2-mediated inflammation and suboptimal antigen presentation^41–44^. Collectively, these taxonomic and functional patterns indicate that microbiome-encoded variation in bacterial cell wall and membrane biosynthesis shapes mucosal immunity and vaccine responsiveness^45^.

This is the first study to analyze the influence of microbiome on vaccine-induced immune responses and vaccine efficacy of an oral ETEC vaccine. The study population is relatively homogeneous, healthy American volunteers with limited or no prior exposure to ETEC. The vaccine efficacy was tested against a well-characterized ETEC challenge strain in a controlled environment within an inpatient unit, thereby reducing confounding factors that could bias the results. This study also has limitations, including a small sample size and reliance on 16S rRNA gene sequencing, which limits taxonomic resolution and precludes direct conclusions about strain-level variation. Functional insights were derived from predictive metagenomics (PICRUSt2) and therefore should be interpreted as hypothesis-generating. The comparative analysis focused on two timepoints for microbiome profiling, which missed any short-term changes in dynamics. Future studies could be carried out with serial sampling and in geographically diverse population incorporating multi-omics approaches (metagenomics, metatranscriptomics, and metabolomics).

This study highlights the intricate relationship between gut microbial ecology and vaccine immunogenicity and protection from colonization and diarrhea. We demonstrate that higher gut microbial α-diversity, while generally considered a hallmark of gut health, was paradoxically associated with reduced mucosal IgA responses to key ETEC antigens, suggesting a potential immunoregulatory effect. In contrast, increased β-diversity reflecting shifts in community structure was positively associated with systemic IgA responses, emphasizing the role of microbial composition in shaping immune priming. Additionally, specific bacterial taxa were found to either enhance or impair vaccine responses and shedding dynamics. These findings underscore the potential for microbiota-informed strategies to enhance the efficacy of oral vaccines.

## Methods

### ETEC vaccine clinical trial and samples

*ETEC vaccine and challenge strain*: ACE527 is a live oral ETEC vaccine composed of three attenuated ETEC strains designed to express a range of ETEC colonization factor antigens (CFA) comprising coli-surface antigens (CS), namely CFA/I, CFA/II (CS1, CS2, CS3), and CFA/IV (CS5, CS6) and the B-subunit of heat-labile enterotoxin (LTB)^46–47^. Studies have demonstrated that the ACE527 vaccine is safe and can elicit significant immune responses to key ETEC antigens^46–48^ H10407 is a wild-type virulent ETEC, serotype O78:H11 that produces both heat-labile (LT) and heat-stable (ST) toxins and expresses colonization factor CFA/I ^46,48^.

### ACE527 vaccine clinical trial design and samples

As reported previously, in a placebo-controlled, double-blind, phase 2b study, 70 healthy American volunteers were enrolled and randomized to receive either the placebo [CeraVacx Buffer (Cera Products, Columbia, MD)] or two doses of ACE527 in CeraVax (each dose approximately 10^11^ CFU of ACE527), 3 weeks apart^46^. Four weeks after their second dose, a total of 27 ACE527 recipients and 27 placebo recipients were challenged with 2 × 10^7^ CFU of H10407 virulent ETEC strain in the inpatient unit of the Centre for Immunization Research (CIR), JHU (Fig. 1). The vaccine was well tolerated and induced robust immune responses to key antigens. Although only a 27% reduction was observed in the primary endpoint, moderate-to-severe diarrhea (MSD), the vaccine significantly reduced intestinal colonization by the challenge strain, as measured by quantitative fecal culture 2 days after challenge, indicating the induction of a functional immune response to the CFA/I antigen. In a post-hoc analysis of additional exploratory diarrhea endpoints, the vaccine protected (41% p = 0.03) against severe diarrhea based on stool volume (> 800 grams of grade 3–5 stools over the inpatient surveillance period). Of note, a later phase 2b trial with three doses of lyophilized ACE527 (~3 × 10^9^ of each strain per dose) administered orally with dmLT adjuvant increased the PE to 65.9% (95% confidence interval [CI] 5.4–87.7, p = 0.003)^49^.

### Samples and data analyzed in the present study

In the present study, since our primary aim was to elicit the association between microbiota with immune responses after vaccination and challenge, we focused only on the ACE527 vaccinees. Samples and data were used from the 27 volunteers who completed two doses of the ACE527 vaccine and were challenged with ETEC H10407 as shown in Fig. 1. For sequencing and analysis of the gut microbiome, stool samples from 27 volunteers at V0 (before the first vaccine dose) and C-1 (day before challenge) were used. Specific microbial taxa associations with immune responses between V0 to V21 (day of the second dose), V0 to V28 (7 days after the second dose) and V0 to V31 (10 days after the second dose) were determined through statistical analysis mentioned in later sections. Stool samples were collected up to three times daily after challenge to monitor shedding of the H10407 challenge strain, with quantitative cultures performed for ACE527 recipients on days 2 and 4 to measure bacterial load as CFU/g stool. In ACE527 vaccinees, a significant reduction was seen in the level of shedding of H10407 in stool on day 2 after challenge, whereas by day 4 their level of shedding approximated that in placebo recipients^46^. Taxon association post-challenge with outcome (MSD), maximum shedding, and length of shedding of challenge strain were determined statistically as described later.

### Vaccine-induced antibody titers data

The immunology methods and data have been reported previously ^46^. The data from the systemic and mucosal antibody responses to potential protective vaccine antigens (LTB, CFA/I), which are also present in the challenge strain, measured by ELISA in serum and ALS were used in this study^47,48^. Serum IgG and IgA responses were evaluated on V0, V21, and V31. Mucosal immune responses were assessed using the ALS assay (for days V0, V21, and V28), which measures IgA secreted by peripheral blood mononuclear cells (PBMC) circulating to mucosal inductive sites, peaking at 7 to 10 days following oral immunization.

### DNA extraction and sequencing

Gut microbiome composition and functional profiles were ascertained from 300mg of fecal specimen collected on V0 and C-1. Total DNA was extracted using the DNeasy^®^ PowerSoil^®^ Pro Kit with bead beating^50^. The V3-V4 region of the 16S rRNA gene was amplified by PCR, barcoded and dual-indexed according to established protocols^51^. DNA was quantified using an Agilent Technologies 2100 Bioanalyzer before pooling. High-throughput next-generation sequencing of the V3-V4 hypervariable region of the 16S rRNA gene was performed using 319 F (5′-GTGCCAGCMGCCGCGGTAA-3′) and 806 R (5′-GGACTACHVGGGTWTCTAAT-3′) universal primers containing a linker sequence required for Illumina MiSeq 250 bp paired-end sequencing. Negative controls were included (n = 2).

### Genome processing, quality control and in silico functional analyses

Sequenced reads were trimmed from forward and reverse adaptor sequences using cutadapt with default settings^52^. Reads were quality-filtered, deduplicated, chimeras removed and amplicon sequence variants (ASV) inferred using *DADA2* ^53^. Forward and reverse reads were merged with 20bp overlap, and taxonomies were assigned using *DADA2* against the Silva database ^53,54^. Contaminants were identified and removed using *decontam* ^55^. ASV abundances were aggregated to the genus level and normalized to relative abundances for statistical analyses. Taxon relative abundances were dichotomized as present or absent if the relative abundance distribution fitted a zero-inflated beta-distribution better than a beta distribution using generalized additive models for location, scale and shape.

We inferred functional profiles from ASV abundances using PICRUSt2^56^. Predicted bacterial metabolic pathway abundances were derived using the MetaCyc database. Pathway abundance contributions were stratified by ASV and aggregated up to the genus level.

### Statistical analysis

Antibody titers against two ETEC antigens *viz*., LTB and CFA/I were transformed into fold changes from V0 to V21, V0 to V28 or V0 to V31, and normalized to a Gaussian distribution prior to analysis^46–48^. We determined the association between diarrhea and microbiota α-diversity (inverse Simpson diversity, Shannon diversity, and Pielou’s evenness) by bias reduced simple logistic regression to accommodate data separation due to the small sample size ^57^. The dependent variable was MSD versus mild or no diarrhea. α-diversity indices were log_2_ transformed. One model was fit for each index. We investigated β-diversity using Bray-Curtis and Jaccard dissimilarity matrices visualized by non-metric multidimensional scaling (NMDS) and compositional associations with diarrhea were tested by microbiome regression-based kernel association test (MiRKAT)^58^. Finally, we identified taxa associated with diarrhea using the relaxed lasso penalized logistic regression, with 10-fold cross validation^59^. A multivariable logistic regression model was fitted using the selected taxa as covariates to estimate odds ratios (OR) and 95% confidence intervals (95%CI) using biased reduced logistic regression. Analyses were repeated as described, but replacing maximum shedding, length of shedding, and antibody titer separately, as the dependent variable, and using linear regression. Maximum shedding was log_10_ transformed prior to analyses.

Individual taxa selected by relaxed LASSO regression as predictors of MSD, shedding or antibody fold change were classified as protective if they predicted decreased odds of diarrhea, reduced shedding, or increase antibody titer fold change; harmful if they predicted increased diarrhea, shedding, or decreased antibody fold change; or as having no associations otherwise. Two-sided Fisher’s Exact tests were performed to identify MetaCyc pathways (predicted by PICRUSt2) that differentiated protective or harmful taxa from others, with adjustment for multiple hypothesis testing to preserve the false discovery rate (FDR)^60^. Thirty-six MetaCyc pathways were differentially present between these three groups of taxa after FDR-adjustment. We performed overrepresentation analyses using one-sided Fisher’s Exact tests to determine whether the differentially present pathways were more likely to have particular biological functions defined by MetaCyc pathway types^61–62^.

Changes in α-diversity or individual taxa pre- to post-vaccination were determined by Wilcoxon signed-rank sum test or McNemar’s test for continuous or dichotomous microbiome features as appropriate.

Statistical significance was determined at α = 0.05. Taxa present in < 10% of specimens were removed prior to statistical analyses. Relative abundances were fitted to beta and zero-inflated beta distributions using *gamlss* ^63^. α-diversity indices were calculated and NMDS was performed using *vegan*^64^. MiRKAT was performed using *MiRKAT*. *glmnet* was used to perform relaxed lasso penalized regression. Biased reduced logistic regression was performed using *brglm2*^65^. Normalization of antibody titer fold changes to a Gaussian distribution was performed using *best-Normalize*. Phylogenic relationships between taxa were visualized using *ggtree*^66^. All analyses were conducted using R version 4.2.2.

## Supplementary Material

Supplementary Files

This is a list of supplementary files associated with this preprint. Click to download.

• SupplementaryFile1.docx

• SupplementaryFile2.pdf

## Figures and Tables

**Table 1:** αdiversity changes between V0 and C-1

Indices	V0 Median [IQR]	C-1 Median [IQR]	p-value
Shannon	2.99 [2.66,3.14]	3.03 [2.87,3.11]	0.693
Inverse Simpson	11.73 [8.79,13.43]	12.57 [9.78,13.83]	0.335
Pielou’s Evenness	0.69 [0.66,0.73]	0.70 [0.67,0.72]	0.757

**Table 2:** Within-subject β-diversity from V0 to C-1

β-diversity index	Median [IQR]	P-value
Within-subject Bray Curtis	0.28 [0.21,0.32]	< 0.001
Within-subject Jaccard	0.44 [0.35,0.48]	< 0.001

## Data Availability

The dataset supporting the findings of this study are available in the ‘Supplementary Files’ accompanying this article. The corresponding author is working with the Johns Hopkins Institutional Review Board (IRB) to determine the extent to which further anonymized individual-level data from human volunteers may be deposited in an appropriate open-access repository in compliance with ethical and regulatory requirements. Pending IRB approval, the data will be made available through a recognized public repository with a persistent identifier.
